# Simulation of a Hemispherical Chamber for Thermal Inkjet Printing

**DOI:** 10.3390/mi13111843

**Published:** 2022-10-28

**Authors:** Xishun Peng, Anjiang Lu, Pangyue Li, Zhongpeng Chen, Ziran Yu, Jianwu Lin, Yi Wang, Yibo Zhao, Jiao Yang, Jin Cheng

**Affiliations:** 1 College of Big Data and Information Engineering, Guizhou University, Guiyang 550000, China; 2 School of Optoelectronic Engineering, Xi’an Technological University, Xi’an 710000, China; 3 Wuxi Imeng Technology Co., Ltd., Wuxi 214000, China

**Keywords:** Inkjet printing, droplet, hemispherical chamber, fluid

## Abstract

It is crucial to improve printing frequency and ink droplet quality in thermal inkjet printing. This paper proposed a hemispherical chamber, and we used the CFD (computational fluid dynamics model) to simulate the inkjet process. During the whole simulation process, we first researched the hemispherical chamber’s inkjet state equipped with straight, conical shrinkage, and conical diffusion nozzles. Based on the broken time and volume of the liquid column, the nozzle geometry of the hemispherical chamber was determined to be a conical shrinkage nozzle with a specific size of 15 µm in height and 15 µm in diameter at the top, and 20 µm in diameter at the bottom. Next, we researched the inkjet performance of the square chamber, the round chamber, and the trapezoidal chamber. The round chamber showed the best inkjet performance using 1.8 µs as the driving time and 10 MPa as the maximum bubble pressure. After that, we compared the existing thermal inkjet printing heads. The results showed that the hemispherical chamber inkjet head had the best performance, achieving 30 KHz high-frequency printing and having the most significant volume ratio of droplet to the chamber, reaching 14.9%. As opposed to the current 15 KHz printing frequency of the thermal inkjet heads, the hemispherical chamber inkjet head has higher inkjet performance, and the volume ratio between the droplet and the chamber meets the range standard of 10–15%. The hemispherical chamber structure can be applied to thermal inkjet printing, office printing, 3D printing, and bio-printing.

## 1. Introduction

The technology of inkjet printers has matured since the 1990s. The two primary inkjet sensors are the thermal inkjet printing head and the piezoelectric inkjet printing head [1,2,3]. Piezoelectric inkjet heads require strict sealing of the inkjet chamber and are more expensive than thermal inkjet heads. In thermal inkjet printing heads, the nozzle geometries are straight, conical shrinkage, and conical diffusion. Most chambers are square, round, or trapezoidal. The material of the heating resistor is TaN film [4,5]. However, thermal inkjet printing is severely limited by its low printing frequency, small ink droplet volume, and unclear handwriting, making it challenging to apply in scientific research [6]. In order to improve the inkjet printing frequency, a hemispherical chamber structure is proposed. In this study, a hemispherical chamber is proposed to refer to the structure of the gradually reduced tube [7,8]. Due to the minification and complexity of inkjet heads [9], a traditional trial-and-error approach and time cost are unsuitable for in-depth research. Therefore, we used the CFD (computational fluid dynamics model) to simulate the entire inkjet process. The CFD model has become essential to the current inkjet print head design in the last few years. Zhou et al. [10] successfully used COMSOL software to study the volume and velocity of ink droplets under different driving voltages and currents. Sohrabi et al. [11] and Tan et al. [12] also simulated the inkjet process using CFD3 and LBM (Lattice Boltzmann methods). In addition, Tan et al. also simulated the ejection of cells from a thermal inkjet print head. Because the CFD model is mature, we used it to simulate the quality of ink droplets and the velocity of the liquid column during the inkjet process. 

**Figure 1:**
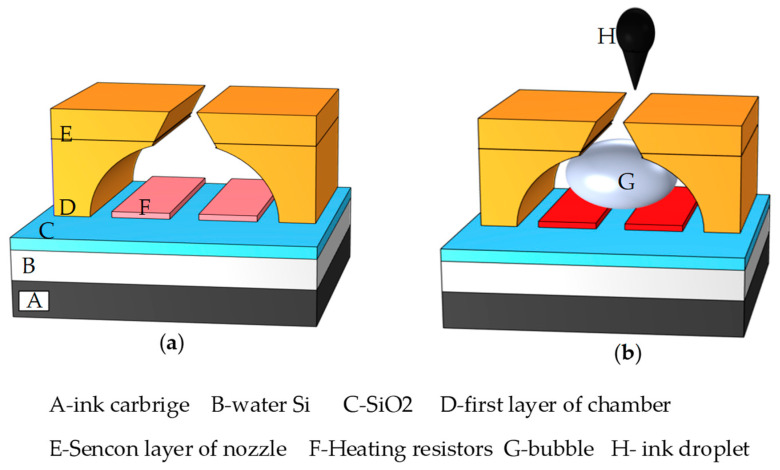
The working process of thermal inkjet head. (**a**) The structure of thermal inkjet head; (**b**) By heating the TaN film, the liquid in the chamber is vaporized to form a vapor bubble, which pushes the liquid out of the nozzle.

As shown in Figure 1, the primary application field of thermal bubble inkjet printing was to eject water-based ink. As shown in Figure 1, based on the rapid heating of the heating resistors, the liquid in the chamber instantly reached more than 600 K, vaporizing and forming a high-pressure bubble. Due to the fluid movement’s inertia, the pressure from this process pushed the fluid around the bubble until it eventually ejected from the nozzle as an ink droplet [13,14].

Since 2009, related scholars have used thermal bubble inkjet printing heads for bioprinting [15,16,17]. For example, Cui et al. [18] printed Chinese hamster ovary cells with a modified HP Deskjet 500 inkjet head and an HP51626A cartridge. In order to promote the application range of thermal inkjet printing heads, it is crucial to improve the droplet volume and the printing frequency of inkjet heads [19]. In this paper, a hemispherical chamber structure is designed using simulation software based on the existing thermal bubble inkjet head structure. Then, the work of a hemispherical chamber equipped with different nozzles is studied. In addition, the influence of contact Angle on ink droplet forming was also studied. The results show that the conical shrinkage nozzle has the best performance. Finally, the specific parameters of the hemispherical thermal inkjet printing head are obtained, and the effectiveness of the modified structure is verified by comparing it with three mainstream structures in the market. 

## 2. Theory and Simulation Model

### 2.1. Steam Bubble Model

In this study, the level-set method is used here to establish a gas–liquid model. The fluid interface is represented by a 0.5 contour line of the level-set function Φ. In air, Φ = 0, and in water, Φ = 1. Accordingly, the level set function is regarded as the water volume fraction. The following equation can describe the fluid interface between the two phases [10]:

(1) $$\frac{\partial \Phi }{\partial t}+u\cdot \nabla \Phi =\alpha \nabla \cdot \left[\varepsilon \nabla \Phi -\Phi \left(1-\Phi \right)\frac{\nabla \Phi }{\left|\nabla \Phi \right|}\right]$$

Parameter ε specifies the interface thickness. When using the numerical stabilization method for the level set equation, the interface thickness can be specified as $\varepsilon =h_{c}/2$, where $h_{c}$ is the typical mesh size in the region passed by the droplet., and α is the most significant value that occurs to the velocity field. Using the N-S (Navier-Stokes) equation, we can research volume and momentum transfer over the interface. As the fluid’s velocity is less than the speed of sound, an incompressible laminar flow is assumed in order to simplify the calculations:

(2) $$\frac{\partial u}{\partial t}+u\cdot \nabla u=-\frac{1}{\rho }\nabla P+\frac{1}{\rho }\nabla \cdot \left[\mu \left(\nabla u+{\left(\nabla u\right)}^{T}\right)\right]+g+f_{sf}\;\nabla \cdot u=0$$

where u is the velocity, *t* is the time, μ is the viscosity, *P* is the pressure, ρ is the density, g is the gravity, and $f_{sf}$ is surface tension.

(3) $$f_{sf}=\sigma \delta k\vec{n}$$

where $\vec{n}$ is the normal interface phase, σ is the surface tension coefficient (N/m), k is the curvature ($k=-\nabla \cdot \vec{n}$) and δ is the Dirac delta function. The normal direction $\vec{n}$ of the interface here is:

(4) $$\vec{n}=\frac{\nabla \Phi }{\left|\nabla \Phi \right|}$$

For function δ:

(5) $$\delta \left(x\right)=0,\;\left(x\neq 0\right)\;\overset{\infty }{\underset{-\infty }{\int }}\delta \left(x\right)dx=1$$

In this study, it can be approximated as:

(6) δ=6|Φ(1−Φ)||∇Φ|

At the interface of the N-S equation, the Heaviside function H(Φ) smoothed the viscosity mutation and density mutation [20]:

(7) $$\rho \left(\Phi \right)=\rho_{air}+\left(\rho_{ink}-\rho_{air}\right)H\left(\Phi \right)$$

(8) $$\mu \left(\Phi \right)=\mu_{air}+\left(\mu_{ink}-\mu_{air}\right)H\left(\Phi \right)$$

In order to study the change in the bubble interface, the Cahn-Hilliard equation is used to track the change in the two-phase flow interface.

(9) $$\frac{\partial \varphi }{\partial t}+u\cdot \nabla \varphi \;=\nabla \cdot \frac{\gamma \lambda }{\varepsilon^{2}}\nabla \psi$$

(10) $$\psi =-\nabla \cdot \varepsilon^{2}\nabla \varphi +\left(\varphi^{2}-1\right)\varphi$$

where φ is the dimensionless phase field variable, (−1≤φ≤1), γ is the mobility (m^3^·s/kg), λ is the mixed energy density (N), and ψ is the phase field help variable. Based on the following equation, the mixing energy density and interface thickness are related to the surface tension coefficient σ:

(11) $$\sigma =\frac{2\sqrt{2}}{3}\frac{\lambda }{\varepsilon }$$

The mobility parameter γ determines the time scale of Cahn-Hilliard diffusion. We set the α *=*$\;\varepsilon^{2}$ to make pressure changes more accurate in the simulation setting. 

According to the phase field method, the volume fractions of the various fluids are as follows:

(12) $$V_{f1}=\frac{1-\phi }{2},\;V_{f1}=\frac{1+\phi }{2}$$

In the current model, $V_{f1}$ represents the volume fraction of ink droplets and $V_{f2}$ represents the volume fraction of air. 

### 2.2. Analysis of Inkjet Chamber

**Figure 2:**
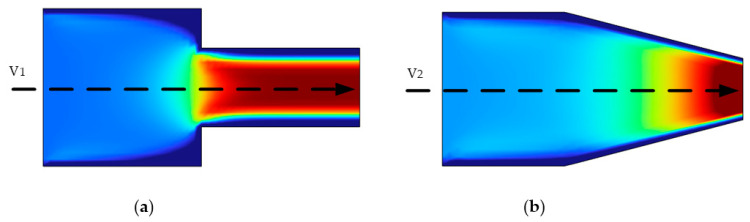
Analyzing the fluid flow in different pipes. (**a**) Analyzing the fluid flow in an abruptly reduced tube; (**b**) Analyzing the fluid flow in a gradually reduced tube.

In this study, a hemispherical chamber is proposed to reduce fluid pressure loss in the chamber. As shown in Figure 2, the fluid flows through the tube and loses some energy because of the viscous effects [21]. The energy loss comprises the frictional pressure loss and the local pressure loss. The energy loss by the liquid to overcome the resistance along the path is called the frictional pressure loss. The energy lost by liquid to overcome local resistance during flow is called local pressure loss.

(13) $$h_{w}=\sum^{\;}h_{f}+\sum^{\;}h_{j}$$

In the above equation, $h_{w}$ is the pressure loss, $h_{f}$ is the frictional pressure loss, and $h_{j}$ is the local pressure loss. In this paper, before designing the inkjet printing structure, the design of suddenly and gradually reduced tubes is referred [22].

According to the definition of fluid mechanics, the local pressure loss in the pipeline is:

(14) $$h_{j}=\zeta \frac{v^{2}}{2g}$$

where ζ is the coefficient of local pressure loss, *v* is the velocity of the fluid, and *g* is the gravity. The local pressure loss coefficient of the gradually reduced tube is usually smaller than that of the suddenly reduced tube. To further increase the printing frequency, increase the droplet volume, and improve pressure utilization rate, we implemented this structure into inkjet printing. In addition, we need to research the filling of the chamber with ink after the ink is finished. Because the liquid level at the nozzle is at the junction of liquid, air and nozzle wall, capillary phenomenon will occur [23,24]. Under capillary force, the ink in the chamber rises fills the whole inkjet head, and finally forms a concave surface at the top of the nozzle.

In order to simplify the analysis difficulty, we choose a section to study the process of the capillary phenomenon. Under one-dimensional flow, the formula of capillary force is as follows [25,26]:

(15) $$h=\frac{2\sigma cos\theta }{\rho gr}$$

where *h* is the height of the rise, σ is the surface tension coefficient, θ is the contact angle, ρ is the liquid density, g is the gravitational acceleration, and *r* is the radius of the capillary. Here, it should be noted that *r* is a fixed value. However, the nozzles researched in this paper include conical shrinkage and conical diffusion nozzles, and their inner diameters are changed, so we differentiate the whole height *h*.

(16) $$\frac{2\sigma cos\theta }{\rho g}=C$$

(17) $$h\;=\Delta h_{1}+\Delta h_{2}+\ldots +\Delta h_{n}$$

(18) $$\Delta h_{1}=C\cdot \frac{1}{r+\Delta r}$$

(19) $$\Delta h_{n}=C\cdot \frac{1}{r+n\Delta r}$$

(20) $$h=C\cdot \overset{n}{\underset{i=1}{\sum }}\frac{1}{r+i\Delta r}$$

Next, this paper will conduct a simulation analysis on the hemispherical chamber and select the current mainstream inkjet printing structure for comparison to verify their inkjet performance.

## 3. Simulation and Results

### 3.1. Hemispherical Chamber Model

**Figure 3:**
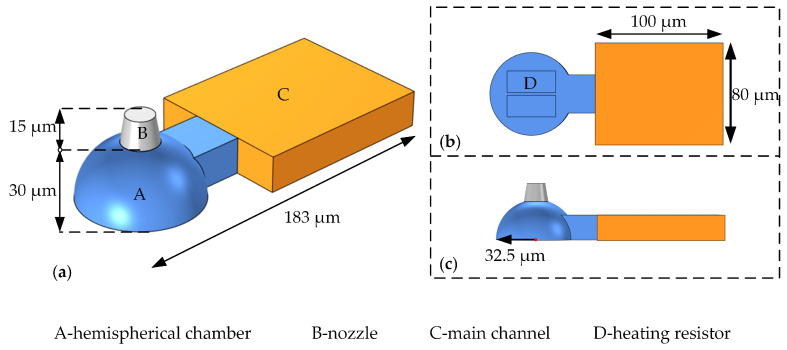
The structural parameters of the hemispherical chamber inkjet head. (**a**) The three-dimensional structure of the hemispherical chamber; (**b**) The side view of the hemispherical chamber; (**c**) The back view of the hemispherical chamber.

In this study, we propose a hemispherical chamber design that will increase ink droplet volume and ensure inkjet performance quality. Figure 3 shows the three-dimensional structure, the back view, and the side view of the hemispherical chamber inkjet head. The design conception came from the vaporization and nucleation of liquid to produce vapor pressure. As the vapor bubble expands, it squeezes the liquid around it and ejects it from the nozzle, pushing a portion of the liquid back into the main channel. For the hemispherical structure, the liquid flows to the nozzle can be more concentrated, and energy can be used more efficiently under similar driving conditions.

The specific size parameters of the hemispherical structure have shown in Table 1. It shows that the inkjet head presented in this study uses a conical shrinkage nozzle with a round hole. The top diameter of the nozzle is slightly smaller than the bottom diameter. The large heating resistors provide the inkjet driving force. The nozzle is located directly above the chamber, facing the resistors. The hemispherical chamber has a radius of 32.5 μm and a height of 30 μm.

**Figure 4:**
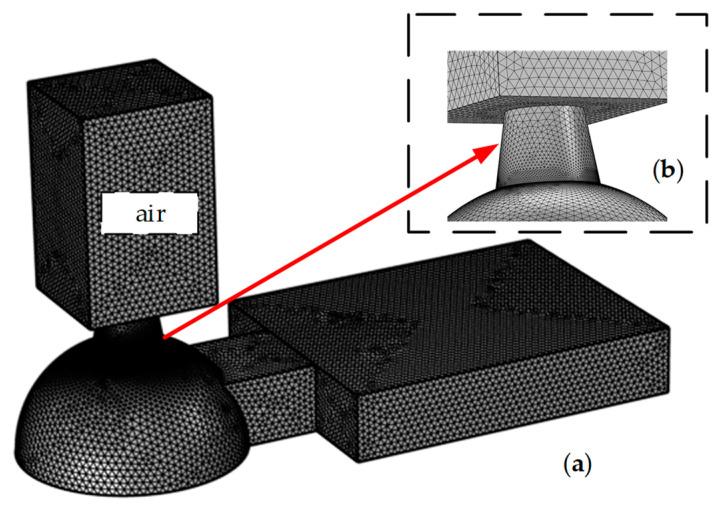
Mesh model of the hemispherical chamber. (**a**) The hemispherical chamber is divided into 952,384 units; (**b**) Enlarged view of nozzle and air domain connection.

When the CFD model is used in simulation research, it is necessary to study the effectiveness of the model. In the previous study, we verified the effectiveness of the simulation model through experiments [6]. Before the inkjet simulation, the meshing rules of the hemispherical chamber were set according to the fluid dynamics, and a 4-hedral structure was used for meshing. Compared with previous studies, as shown in Figure 4, the grid we divided here is more detailed, with a total of 952,384 grids. In this way, the accuracy of prediction is improved [27]. Since the size of the inkjet printing head belongs to the micron level, we adopt 2000 as the set value of the Reynolds number, assuming that the flow state of the inkjet process is laminar flow. The TaN film at the bottom of the chamber uses high-pressure bubbles instead of the thermal simulation [19]. To simulate the bubble inkjet simulation, we refer to the pressure settings in previous papers [6,10,28]. The maximum vapor pressure of the bubble is set to 10 MPa. The liquid viscosity was set as 5 cps, the surface tension was set as 0.4 N/m, and the density was set as 1000 kg/m^3^. The drive time is divided into two periods. The first section is 0–0.1 µs, and the second section is 1.2–1.8 µs. The back of the main channel is set as the open interface, the top of the nozzle is set as the outlet, and the boundary condition of the entire inkjet printing head is set as no slip. In addition, it is essential to consider both the hydrophilicity and the hydrophobicity of the material used for the inkjet head chamber. A direct reflection of the material property is the contact angle between the liquid and the interior wall of the nozzle. The contact angle will determine how long it takes for the droplet to form [29,30]. Generally speaking, the better the hydrophilicity of the material, the smaller the contact angle, and the more apparent the capillary phenomenon. Contact angles were set to 30°, 50°, 70°, 90°, 110° and 130°, respectively.

**Figure 5:**
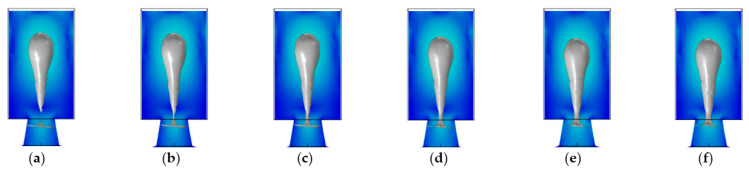
Fracture state of liquid column at different contact angles. (**a**) The contact angle is 30°; (**b**) The contact angle is 50°; (**c**) The contact angle is 70°; (**d**) The contact angle is 90°; (**e**) The contact angle is 110°; (**f**) The contact angle is 130°.

**Figure 6:**
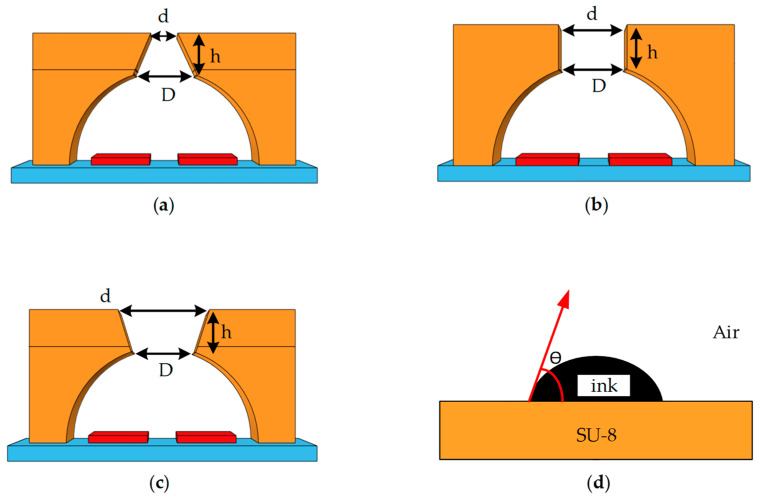
Inkjet heads with different nozzles and the contact angle of the SU-8 chamber. (**a**) Inkjet head equipped with conical shrinkage nozzle; (**b**) Inkjet head equipped with straight nozzle; (**c**) Inkjet head equipped with conical diffusion nozzle; (**d**) The contact angle of the SU-8 chamber.

As shown in Figure 5, the contact angle’s size determined the liquid column’s tensile and broken states. Smaller contact angles had a stronger adhesion to the ink, breaking the liquid column faster and causing the ink droplet to form more rapidly. As the contact angle increased, the tension between the liquid column’s tail and the nozzle’s liquid surface also weakened. Therefore, at 3.8 µs time, the liquid column had broken and formed the ink droplet when the contact angle was 30°. In contrast, when the contact angle was 130°, the tail of the liquid column was thicker and could not reach the fracture standard. At 50° and 70° contact angles, respectively, the tail stretch degree of the liquid column was close, and there was no significant difference. Choosing a material for the chamber also requires considering the material’s electrical conductivity. The liquid is stored in the chamber’s interior, which means electrically conductive materials cause the liquid to ionize. The stable chemical properties of SU-8 photoresist have made it popular among current scholars working in inkjet printing [31]. Its contact angle is 76°. Consequently, we selected the SU-8 photoresist to fabricate the chamber. The nozzle geometry of an inkjet printing head can be classified as conical shrinkage, straight, or conical diffusion. Figure 6 shows the nozzle’s shape and the liquid’s contact angle in the SU-8 chamber. In the next step, we will analyze the motion state of liquid under different nozzle geometries to verify the structure’s size. In the same way, inkjet heads equipped with three types of nozzles are driven under the same conditions. There are three variables: the nozzle’s top, height, and bottom diameter. It was previously stated in [6] that the nozzle height was the same as the chamber thickness, improving manufacturing efficiency and reducing costs. As the inner wall of the hemispheric chamber is curvilinear and slightly higher, the nozzle height is temporarily set at 15 μm.

### 3.2. Analysis of Inkjet Performance

**Figure 7:**
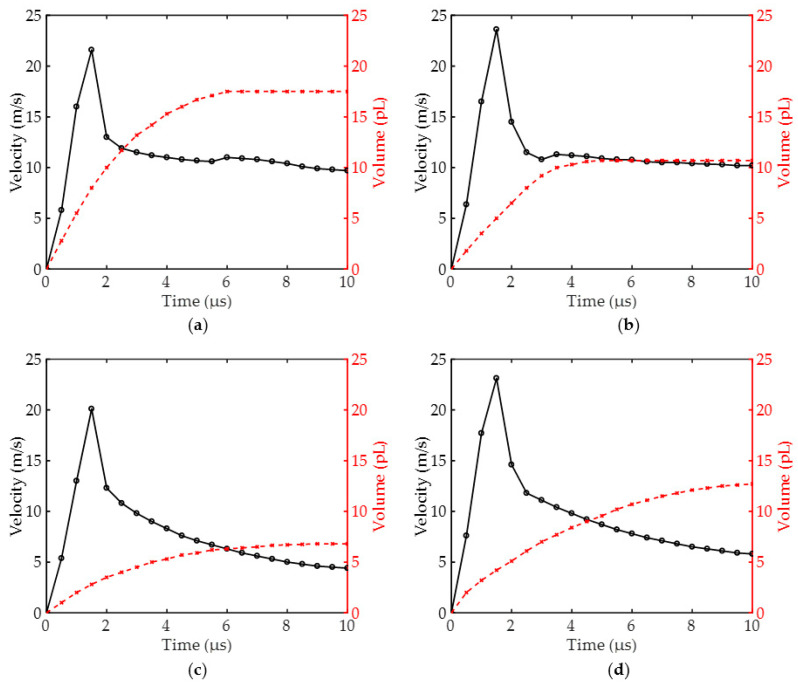
Under different nozzles, the liquid column’s maximum velocity and the droplet’s volume change. (**a**) The liquid column’s velocity and the droplet’s volume change for the straight nozzle; (**b**) The liquid column’s velocity and the droplet’s volume change for the conical shrinkage nozzle; (**c**) When the bottom diameter of the conical diffusion nozzle is 10 μm, the liquid column’s velocity and the droplet’s volume change in conical diffusion nozzle; (**d**) When the bottom diameter of the conical diffusion nozzle is 15 μm, the liquid column’s velocity and the droplet’s volume change in conical diffusion nozzle.

Firstly, we set the top and bottom diameter of the straight nozzle to 20 μm and 20 μm, and the top and bottom diameters of the conical shrinkage nozzle to 15 μm and 20 μm, respectively. Two conical diffusion nozzles are present: the top nozzles are 20 μm, and the bottom diameter is 10 μm and 15 μm, respectively. As shown in Figure 7, we demonstrated the motion state of the liquid under different nozzles before 10 μs, as well as the maximum velocity of the liquid column and the volume of the droplet ejected from above the nozzle. As shown in Figure 7a, in the straight nozzle, the maximum velocity of the liquid column was 21.6 m/s, and the maximum liquid volume above the nozzle was 17.5 pL (pL = 10^−15^ L). In Figure 7b, in the conical shrinkage nozzle, the maximum velocity of the liquid column was 23.6 m/s, and the maximum liquid volume above the nozzle was 10.7 pL. In Figure 7c, when the bottom diameter of the conical diffusion nozzle was 10 μm, the maximum velocity at the head of the liquid column was 20.1 m/s, and the maximum volume of the liquid column above the nozzle was 6.8 pL. In Figure 7d, when the bottom diameter of the conical diffusion nozzle was 15 μm, the maximum velocity at the head of the liquid column was 23 m/s, and the maximum volume of the liquid column above the nozzle was 12.8 pL.

**Figure 8:**
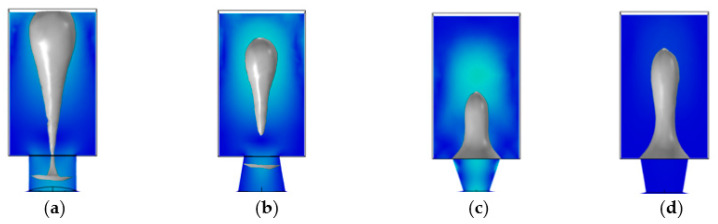
The broken state of the liquid column under different nozzles. (**a**) The broken state of the liquid column in the straight nozzle; (**b**) The broken state of the liquid column in the conical shrinkage nozzle; (**c**) The liquid column was broken when the conical diffusion nozzle’s bottom diameter was 10 μm; (**d**) The liquid column was broken when the conical diffusion nozzle’s bottom diameter was 15 μm.

Furthermore, we need to research the broken time of the liquid column, which directly affects the basic properties of inkjet printing. Figure 8 shows the stretching state of a liquid column under different nozzles. The broken time of the liquid column with a straight nozzle was 6 μs, which formed a large ink droplet. In the conical shrinkage nozzle, the broken time of the liquid column was 4 μs. Nevertheless, in the conical diffusion nozzle, the liquid column did not break before 10 μs, and the maximum velocity of the ink column decreased to 5 m/s. As a result, an ink droplet was not formed at this time. Therefore, we can find that the inkjet performance of straight nozzle and conical shrinkage nozzle is good, while the conical diffusion nozzle is challenging to form an ink droplet in a short time.

**Figure 9:**
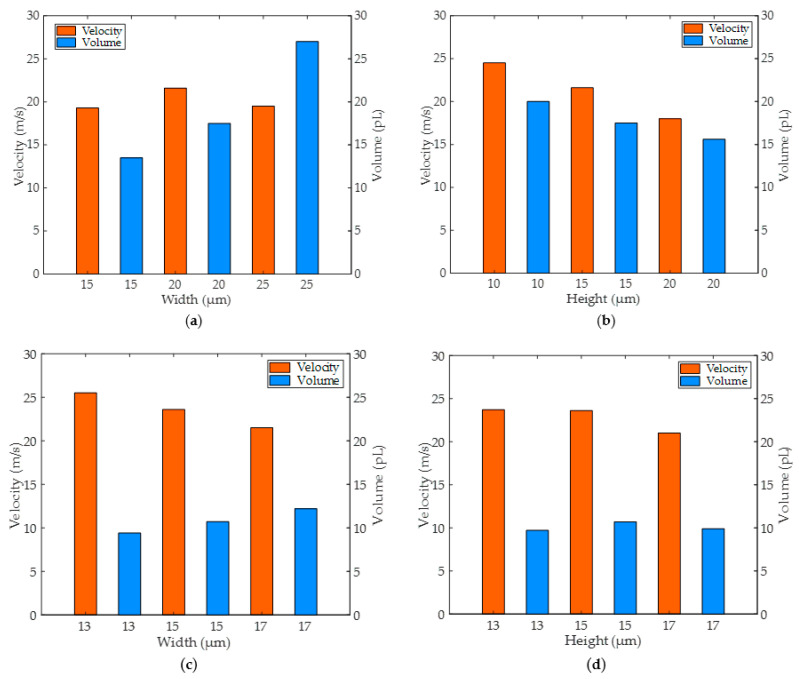
The liquid column’s maximum velocity and the droplet’s volume change for straight and conical shrinkage nozzles. (**a**) Under the different widths of the straight nozzles, the liquid column’s velocity and the droplet’s volume change; (**b**) Under the different heights of the straight nozzles, the liquid column’s velocity and the droplet’s volume change; (**c**) Under the different widths of the conical shrinkage nozzles, the liquid column’s velocity and the droplet’s volume change; (**d**) Under the different heights of the conical shrinkage nozzles, the liquid column’s velocity and the droplet’s volume change.

Next, we continue to study the straight nozzle and the conical shrinkage nozzle. As shown in Figure 9a, the straight nozzle was set at 15 μm height to research fluid motion under various widths. With nozzle widths of 15 μm, 20 μm, and 25 μm, the maximum velocity of the liquid column head was 19.3 m/s, 21.6 m/s, and 19.5 m/s, and the maximum volume of the corresponding liquid column was 13.5 pL, 17.5 pL, and 27 pL, respectively. As the nozzle width increased, the volume of the liquid column above the nozzle increased as well, and the velocity of the liquid column reached the maximum when the diameter was 20 μm. Furthermore, when the nozzle width was 20 μm, we researched the fluid movement state in the straight nozzles at various heights. As shown in Figure 9b, with nozzle heights of 10 μm, 15 μm, and 20 μm, the maximum velocity of the liquid column head was 24.5 m/s, 21.6 m/s, and 18 m/s, and the maximum volume of the corresponding liquid column was 20 pL, 17.5 pL, and 15.6 pL, respectively. As the straight nozzle height increased, both the maximum velocity of the liquid column head and its volume decreased. On the surface, we should select the size of the liquid column with the maximum velocity and volume. However, we must consider the accuracy of inkjet printing and the length of the entire printing period. It will reduce printing clarity when the size of the ink droplet is small, but it will affect accuracy if the droplet size is too large. Moreover, as the volume of the liquid column increases, the amount of ink that needs to be refilled for the next inkjet print also increases, increasing the filling time. Thus, we select a straight hole nozzle with a 20 μm width and 15 μm height.

It is also essential to research the conical shrinkage nozzle, as shown in Figure 9c. With a height of 15 μm and a bottom diameter of 20 μm, the top diameters of the nozzles were 13 μm, 15 μm, and 17 μm. The maximum velocity of the liquid column head was 25.5 m/s, 23.6 m/s, and 21.5 m/s, respectively, and the maximum volume of the liquid column above the nozzle was 9.4 pL, 10.7 pL, 12.2 pL. We found that with the top diameter of the conical shrinkage nozzle increasing, the maximum velocity of the liquid column head decreased as its volume increased. Here, the nozzle with a bottom diameter of 13 μm ejected the liquid column fastest. After the ink droplet was broken, the jitter range became more significant, and the ejected liquid column did not reach 10 pL in volume [32]. It is important to note that a nozzle with a top diameter of 17 μm ejects a slower liquid column with a larger volume, further prolonging the ink filling time. As a result, we choose a nozzle with a top diameter of 15 μm. As shown in Figure 9d, in order to further research the nozzle height, we set the heights to 13 μm, 15 μm, and 17 μm, respectively. The maximum velocity of the liquid column head was 23.7 m/s, 23.6 m/s, and 21 m/s, respectively, and the maximum volume of the ejected liquid column was 9.7 pL, 10.7 pL, and 9.9 pL. We can find that the velocity of the liquid column head decreases gradually as nozzle height increases, and the volume of the liquid column does not increase here, which is an interesting phenomenon. As the nozzle height increases, the fluid form a local high pressure inside the nozzle, and the ejecting liquid column gets inertia to action. Thus, the ink droplet is broken earlier, and the ink droplet’s volume will decrease. In the end, we choose the nozzle height of 15 μm. Based on the results above, we will simulate the inkjet process using straight and conical shrinkage nozzles. For the straight nozzle, the width is 20 μm, and the height is 15 μm. For the conical shrinkage nozzle, the top diameter is 15 μm, the bottom diameter is 20 μm, and the height is 15 μm.

**Figure 10:**
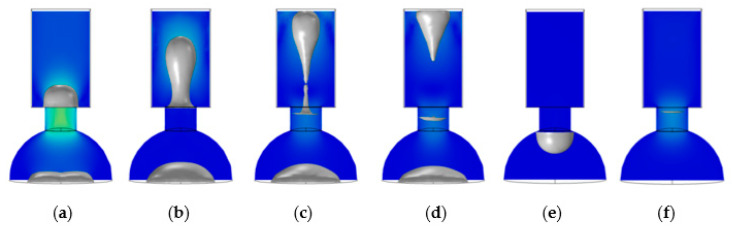
Inkjet process with the straight nozzle. (**a**) At 1 μs, the nozzle began to squeeze out the liquid; (**b**) At 4 μs, the liquid column formed; (**c**) At 6 μs, the liquid column was broken, and the droplet was formed; (**d**) At 8 μs, the droplet flew out from the air domain; (**e**) At 20 μs, the bubble disappeared in the chamber; (**f**) At 46 μs, the chamber was already filled.

In Figure 10a, at 1 μs, the nozzle began to squeeze out the liquid. Due to the drive time of 2 μs, as shown in Figure 10b, the liquid column began to stretch and taper off at 4 μs. In Figure 10c, the liquid column was broken, an ink droplet formed at this time, and the corresponding time was 6 μs. In Figure 10d, at 8 μs, the tail of the droplet gradually merged with the head, and the distance between the tail and head decreased as it flew in the air domain. In Figure 10e, at 20 μs, the bubble disappeared in the chamber. In Figure 10f, at 46 μs, the liquid rose upward under the capillary action, forming a concave surface in the chamber wall and nozzle.

**Figure 11:**
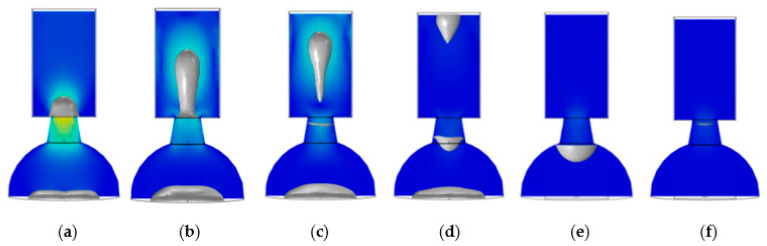
Inkjet process with the conical shrinkage nozzle. (**a**) At 1 μs, the nozzle began to squeeze out the liquid; (**b**) At 3 μs, the liquid column formed; (**c**) At 4 μs, the liquid column was broken, and the droplet was formed; (**d**) At 7 μs, the droplet flew out from the air domain; (**e**) At 15 μs, the bubble disappeared in the chamber; (**f**) At 33 μs, the chamber was filled already.

As shown in Figure 11a, at 1 μs, due to the smaller diameter at the top of the nozzle, the conical shrinkage nozzle produced a smaller volume of the liquid column than the straight nozzle. As shown in Figure 11b, the liquid column began to stretch at 3 μs, and the tail gradually tapers off. In Figure 11c, at 4 μs, the ink droplet was formed when the liquid column tail was broken with the liquid level. As shown in Figure 11d, at 7 μs, the droplet merged further, and the distance between the head and tail decreased significantly, leaving the air domain. In Figure 11e, the bubble disappeared in the chamber after 15 μs, leaving a concave surface at the bottom of the nozzle. At 33 μs, as shown in Figure 11f, the liquid inside the nozzle is replenished enough to form a concave surface. The gravity and surface tension of liquid mainly caused the formation of concave surfaces here. Finally, based on the results of Figure 10 and Figure 11, the nozzle geometry is determined to be a conical shrinkage nozzle with a 15 μm top diameter, 20 μm bottom diameter, and 15 μm height.

**Figure 12:**
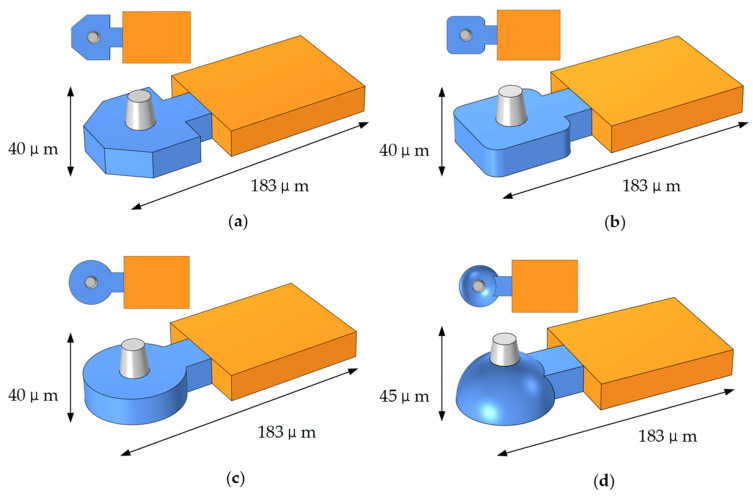
Inkjet head parameters for different chambers. (**a**) Structural parameters of the trapezoidal inkjet head; (**b**) Structural parameters of the square inkjet head; (**c**) Structural parameters of round the inkjet head; (**d**) Structural parameters of the hemispherical inkjet head.

Currently, HP, Epson, and Canon are the three leading thermal inkjet print head manufacturers. According to the existing market research, we categorize the current thermal bubble inkjet printing head into three types: square, trapezoidal, and round. As shown in Figure 12, the hemispherical chamber proposed in this study will be analyzed along with three other types of inkjet print heads. The current mainstream structure has a chamber thickness of 20 μm and a nozzle height of 20 μm. To compare inkjet performance between the four structural chambers, we limit the volume of the chambers to 72 pL.

Table 2 compares four inkjet heads with different structures under the same driving conditions. For the square inkjet head, the maximum velocity of the liquid column was 21 m/s. At 5.6 μs, the liquid column was broken, forming an 8.8 pL ink droplet, and the volume ratio of the ink drop to the chamber was 12.7%. For the trapezoidal inkjet head, the maximum velocity of the liquid column was 21.3 m/s. At 5.4 μs, the liquid column was broken, forming a 9.0 pL ink droplet, and the volume ratio of the ink drop to the chamber was 12.5%. For the round inkjet head, the maximum velocity of the liquid column was 22.1 m/s. At 5.1 μs, the liquid column was broken, forming a 9.2 pL ink droplet, and the volume ratio of the ink drop to the chamber was 12.2%. For the hemispherical inkjet head, The maximum velocity of the liquid column was 23.6 m/s. At 4 μs, the liquid column was broken, forming a 10.7 pL ink droplet, and the volume ratio of the droplet to the chamber was 14.9%.

**Figure 13:**
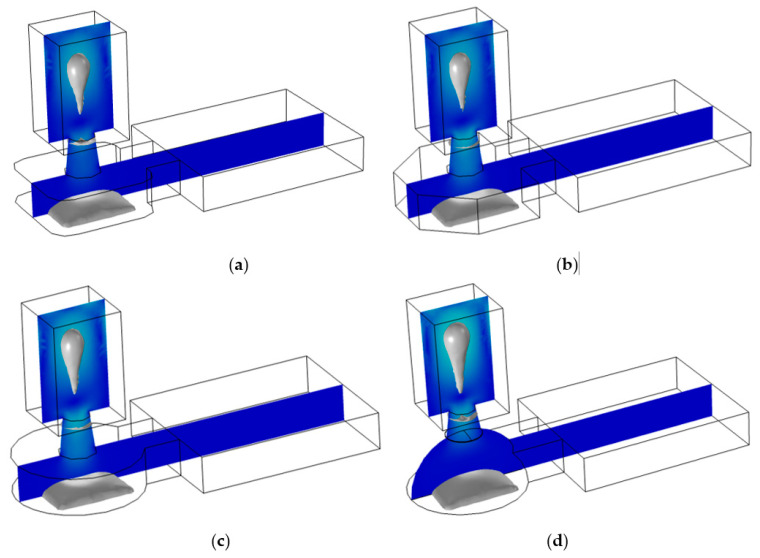
The volume of droplets under different inkjet heads. (**a**) For the square inkjet head, the droplet volume is 8.8 pL; (**b**) For the trapezoidal inkjet head, the droplet volume is 9.0 pL; (**c**) For the round inkjet head, the droplet volume is 9.2 pL; (**d**) For the hemispherical inkjet head, the droplet volume is 10.7 pL.

As a result of the research presented in Table 2, the hemispherical inkjet head performed best, and the maximum ejection velocity of the liquid column was consistent with the experimental data given in [19]. Additionally, we showed three-dimensional images of liquid columns broken under four structure inkjet heads. As shown in Figure 13d, it could be seen that the volume of the ink droplet ejected by the hemispherical inkjet head was more remarkable than the other three existing inkjet heads. The maximum droplet volume was 10.7 pL, or 14.9% of the total chamber volume, which was in line with the current industry accepted range of 10% to 15% [6,10,11].

Additionally, we compared the total inkjet time of the four inkjet heads. The inkjet period of the square, trapezoidal, circular, and hemispherical inkjet heads was 46 μs, 42 μs, 40 μs, and 33 μs, respectively. Based on the results, the round inkjet head has the largest ink droplet volume and the shortest inkjet time among the three traditional inkjet heads. Compared with the round inkjet head, the hemispherical chamber inkjet head proposed in this study increased the droplet volume by 16.3% and reduced the inkjet period by 13.9%. In order to evaluate the performance of the hemispherical chamber inkjet head, we compare it with existing thermal inkjet printing heads. 

As shown in Table 3, we proposed a hemispherical chamber inkjet printing head with the shortest broken time of the liquid column, only 4 μs, and the liquid column velocity was the fastest at 23.6 m/s. The printing frequency was the highest, reaching 30 KHz. The volume ratio of droplet to cavity reached 14.9%, meeting the standard of 10–15%. Based on the above results, the performance of inkjet printing is higher than other existing inkjet heads.

## 4. Conclusions

In this study, we use the CFD simulation model to study the inkjet printing process and propose a structure of the hemispherical chamber. There were approximately 72 pL of volume within the hemispherical chamber, which measured 32.5 μm in diameter and 30 μm in height. As a result of the simulation analysis, we determined that the top diameter of the nozzle was 15 μm, the bottom diameter was 20 μm, and the height of the nozzle was 15 μm. Based on a driving time of 2 μs and a maximum bubble pressure of 10 MPa, the maximum velocity of the liquid column was 23.6 m/s. In addition, a 10.7 pL ink droplet volume formed and occupied 14.9% of the chamber volume, and the inkjet period was 33 μs. Under the same driving conditions, the parallel structures, square, trapezoidal, and round of the current mainstream were researched. Among the three structures, the round inkjet head had the best performance. The maximum velocity of the liquid column was 22.1 m/s, the fracture time was 5.1 μs, the droplet volume was 9.2 pL, and the inkjet period was 40 μs. By comparison with round inkjet heads, hemispherical heads had a 16.3% higher droplet volume and a 13.9% shorter inkjet time, which reached the threshold of 30 KHz. In the end, under the same simulation parameters, we compared a variety of existing thermal inkjet heads, the proposed hemispherical chamber inkjet head had the best performance, which could achieve high-frequency printing at 30 KHz, and the volume ratio of droplet to cavity reached 14.9%. The hemispherical chamber structure design is suitable for inkjet printing and provides a new structural reference for office printing, 3D printing, and bio-printing equipment.

## Figures and Tables

**Table 1:** The design parameters of the hemispherical inkjet head.

Structural Parameters	Value (µm)
Nozzle top diameter	15
Nozzle bottom diameter	20
The nozzle height	15
Heating resistor	17 × 38
Main channel	80 × 100
Chamber height	30
Chamber radius	32.5

**Table 2:** Comparison of inkjet heads with different chambers.

Chamber Types	Liquid Column Broken Time (μs)	Droplet Volume μm (pL)	Velocity of Liquid Column (m/s)	Volume Ratio of Droplet to Chamber (%)
square	5.6	8.8	21.0	12.2
trapezoidal	5.4	9.0	21.3	12.5
round	5.1	9.2	22.1	12.7
hemispherical	4	10.7	23.6	14.9

**Table 3:** Comparison of different shapes of inkjet heads.

Reference	Liquid Column Broken Time (μs)	Velocity of Liquid Column (m/s)	Maximum Bubble Pressure (MPa)	Nozzle Diameter (μm)	Printing Frequency (KHz)
Ref. [11]	22	21	20	48	-
Ref. [12]	15	16	-	20	-
Ref. [27]	61	-	5.5	50	-
Ref. [32]	-	6	-	20	1
Ref. [33]	15	16	2	20	-
This work	4	23.6	10	20	30

## Data Availability

Not applicable.
